# The role of global longitudinal strain change in the assessment of early cardiotoxicity in patients receiving immune checkpoint inhibitors: a prospective pilot study of 30 patients

**DOI:** 10.1186/s44348-026-00107-3

**Published:** 2026-11-10

**Authors:** Cem Korucu, Serkan Gökaslan

**Affiliations:** 1 Department of Cardiology, Suruç State Hospital, Şanlıurfa, Türkiye; 2 Department of Cardiology, Afyonkarahisar Health Sciences University, Afyonkarahisar, Türkiye

**Keywords:** Immune checkpoint inhibitors, Global longitudinal strain, Speckle-tracking echocardiography, Cardiotoxicity, Myocarditis

## Abstract

**Background:**

Immune checkpoint inhibitors (ICIs) improve survival in various malignancies but may cause immune-mediated cardiotoxicity. Because left ventricular ejection fraction (LVEF) typically declines late, more sensitive markers are needed for early detection of subclinical myocardial injury. This study investigated the association between changes in global longitudinal strain (GLS) and early cardiotoxicity in patients receiving ICIs.

**Methods:**

In this prospective, single-center pilot study, 30 consecutive adult patients scheduled for ICI therapy were enrolled. Serial transthoracic echocardiography was performed at baseline, 6 to 8 weeks, and 12 weeks to measure LVEF using the biplane Simpson method and GLS using 2D speckle-tracking echocardiography. Cardiotoxicity was defined as a ≥ 10% absolute reduction in LVEF to < 50% and/or new-onset heart failure, suspected myocarditis, clinically significant arrhythmia, or major cardiovascular events. A relative GLS reduction ≥ 15% was considered indicative of subclinical dysfunction.

**Results:**

During a mean 12-week follow-up, cardiotoxicity occurred in six patients (20.0%). In affected patients, GLS significantly deteriorated early (from − 18.2% ± 2.5% to − 14.1% ± 2.8%, *P* < 0.001), preceding any significant decline in LVEF. Patients without cardiotoxicity showed stable GLS and LVEF values. A ≥ 15% GLS reduction was associated with cardiotoxicity and showed exploratory discriminatory ability, with 83.3% sensitivity and 91.7% specificity (area under the curve, 0.86; 95% confidence interval, 0.72–0.97).

**Conclusions:**

Early GLS deterioration was associated with cardiotoxicity despite relatively preserved LVEF. Given the temporal overlap between GLS assessment and cardiotoxicity events in several patients, these findings should be considered exploratory and hypothesis-generating.

## Background

Immune checkpoint inhibitors (ICIs) have fundamentally transformed the therapeutic landscape of multiple malignancies by enhancing antitumor immune responses and significantly improving survival outcomes [1, 2]. Agents targeting programmed cell death protein 1 (PD-1), programmed death ligand 1 (PD-L1), and cytotoxic T-lymphocyte–associated antigen 4 (CTLA-4) are now widely incorporated into standard oncologic treatment algorithms. Despite their well-established clinical efficacy, ICIs are associated with a unique spectrum of immune-related adverse events, among which cardiovascular toxicity, although relatively uncommon, carries substantial morbidity and mortality [3].

ICI-associated cardiotoxicity most commonly manifests as myocarditis, but may also include left ventricular systolic dysfunction, heart failure, arrhythmias, and pericardial disease. Notably, ICI-related myocarditis is characterized by an early onset, fulminant clinical course, and high fatality rates compared with other forms of cancer therapy–related cardiac injury [4, 5]. Early identification of subclinical myocardial involvement is therefore of critical importance for timely initiation of cardioprotective strategies and optimization of oncologic treatment continuity.

Left ventricular ejection fraction (LVEF), the conventional parameter used to define cardiotoxicity, often remains preserved until relatively late stages of myocardial injury, limiting its sensitivity for early detection. Speckle-tracking echocardiography–derived global longitudinal strain (GLS) has emerged as a more sensitive marker of subclinical myocardial dysfunction across various cardiotoxic cancer therapies, particularly in anthracycline- and trastuzumab-treated populations [6–8]. However, data regarding the role of GLS in predicting early cardiotoxicity among patients receiving ICIs remain limited, and prospective evidence is scarce [9]. Although GLS deterioration during ICI therapy has been reported in several retrospective and prospective studies, the magnitude and temporal pattern of these changes remain inconsistent across studies. In particular, some studies have demonstrated early GLS deterioration despite preserved LVEF, whereas others have not observed significant longitudinal changes in GLS.

Given the growing use of ICIs and the potentially life-threatening nature of associated cardiac complications, identifying reliable, noninvasive, and reproducible early markers of myocardial injury is of paramount clinical relevance. Therefore, the present prospective pilot study aimed to investigate the association between longitudinal changes in GLS and cardiotoxicity and to explore the discriminatory ability of early GLS changes in patients undergoing ICI therapy.

## Methods

Patients were prospectively recruited from a single-center cardio-oncology referral population between January 2024 and October 2025. Patients receiving ICI therapy who underwent baseline cardiovascular assessment and met the predefined eligibility criteria were considered for inclusion. Patients with previously documented left ventricular systolic dysfunction (LVEF < 50%), prior exposure to cardiotoxic chemotherapy within the last 6 months, moderate-to-severe valvular heart disease, persistent atrial fibrillation, uncontrolled systemic illness, or inadequate echocardiographic image quality for strain analysis were excluded. Baseline demographic characteristics, cardiovascular risk factors, cancer subtype, and oncologic treatment regimens were systematically recorded prior to ICI initiation. Detailed oncologic treatment information, including ICI regimen, concomitant chemotherapy, targeted therapy, and radiotherapy, was systematically recorded. Particular attention was given to recent or concomitant exposure to potentially cardiotoxic therapies, including anthracyclines and thoracic radiotherapy. All patients underwent standardized clinical evaluation and transthoracic echocardiography at baseline, at 6 to 8 weeks after treatment initiation, and at 12 weeks, or earlier if cardiac symptoms occurred. Echocardiography was performed at baseline and at 6 to 8 weeks, with additional assessments performed earlier when clinically indicated by cardiac symptoms or suspected cardiotoxicity. The timing of echocardiographic assessment relative to the diagnosis of cardiotoxicity was recorded for each patient.

Echocardiographic examinations were performed using a commercially available ultrasound system (Vivid E95, GE HealthCare) equipped with a phased-array transducer, in accordance with current guideline recommendations. Images were acquired in the left lateral decubitus position with electrocardiographic gating. LVEF was calculated using the biplane Simpson method from apical four- and two-chamber views. GLS analysis was conducted offline using dedicated speckle-tracking software (EchoPAC ver. 203, GE HealthCare). Standard apical long-axis, four-chamber, and two-chamber images were obtained at frame rates between 50 and 80 frames per second. Endocardial borders were manually traced at end-systole, automated tracking quality was visually verified, and segments with inadequate tracking were excluded according to guideline-based recommendations. GLS was defined as the average peak systolic longitudinal strain of the 17-segment left ventricular model. Relative GLS change from baseline was calculated as the percentage difference between follow-up and baseline measurements. All echocardiographic analyses were performed by two experienced cardiologists blinded to clinical outcomes, and intraobserver variability was assessed in a randomly selected subset of 15 patients.

Cardiotoxicity was defined according to contemporary cardio-oncology criteria as a new ≥ 10% absolute decline in LVEF to a value < 50%, or the development of clinically significant heart failure, clinically suspected myocarditis, significant ventricular arrhythmia, or another major cardiovascular event during follow-up [10]. For suspected ICI-associated myocarditis, the diagnosis was based on the presence of new cardiac troponin elevation together with compatible clinical and/or imaging findings. Cardiac magnetic resonance imaging and endomyocardial biopsy were not systematically performed in all patients. In addition, subclinical myocardial dysfunction was defined as a relative reduction of ≥ 15% in GLS compared with baseline. The primary endpoint of the study was the development of cardiotoxicity during the 12-week follow-up period, while secondary analyses evaluated temporal changes in GLS and LVEF and the exploratory discriminatory ability of GLS reduction in relation to cardiotoxicity.

Cardiac biomarkers, including high-sensitivity cardiac troponin and N-terminal pro–B-type natriuretic peptide (NT-proBNP), were assessed at baseline and during follow-up at the time of scheduled clinical evaluations. Additional measurements were obtained when clinically indicated or when cardiotoxicity was suspected. Troponin elevation was defined as a value above the assay-specific upper reference limit, whereas an NT-proBNP increase was defined as a > 30% increase from baseline.

Continuous variables were tested for normality using the Kolmogorov–Smirnov test and are presented as mean ± standard deviation or median (interquartile range), as appropriate, whereas categorical variables are expressed as counts and percentages. Comparisons between patients with and without cardiotoxicity were performed using the independent samples t-test or Mann–Whitney U-test for continuous variables and the chi-square test or Fisher exact test for categorical variables. Paired measurements were analyzed using the paired t-test or Wilcoxon signed-rank test. Receiver operating characteristic (ROC) curve analysis was performed to assess the discriminatory ability of relative GLS reduction for cardiotoxicity. For the exploratory ROC analysis, the relative change in GLS from baseline to the 6- to 8-week echocardiographic assessment was evaluated. A relative GLS reduction of ≥ 15% from baseline was prespecified as the threshold for clinically relevant GLS deterioration. Sensitivity, specificity, positive predictive value, negative predictive value, and area under the curve (AUC) were calculated with 95% confidence intervals (CIs). Given the small number of cardiotoxicity events, diagnostic performance estimates were considered exploratory. Intraobserver and interobserver reproducibility of GLS measurements were assessed in a randomly selected subset of 15 patients. Reproducibility was evaluated using intraclass correlation coefficients (ICCs) and Bland–Altman analysis. The second observer was blinded to the measurements of the first observer. A two-sided *P*-value of < 0.05 was considered statistically significant. All statistical analyses were conducted using IBM SPSS ver. 26.0 (IBM Corp).

## Results

**Fig. 1:**
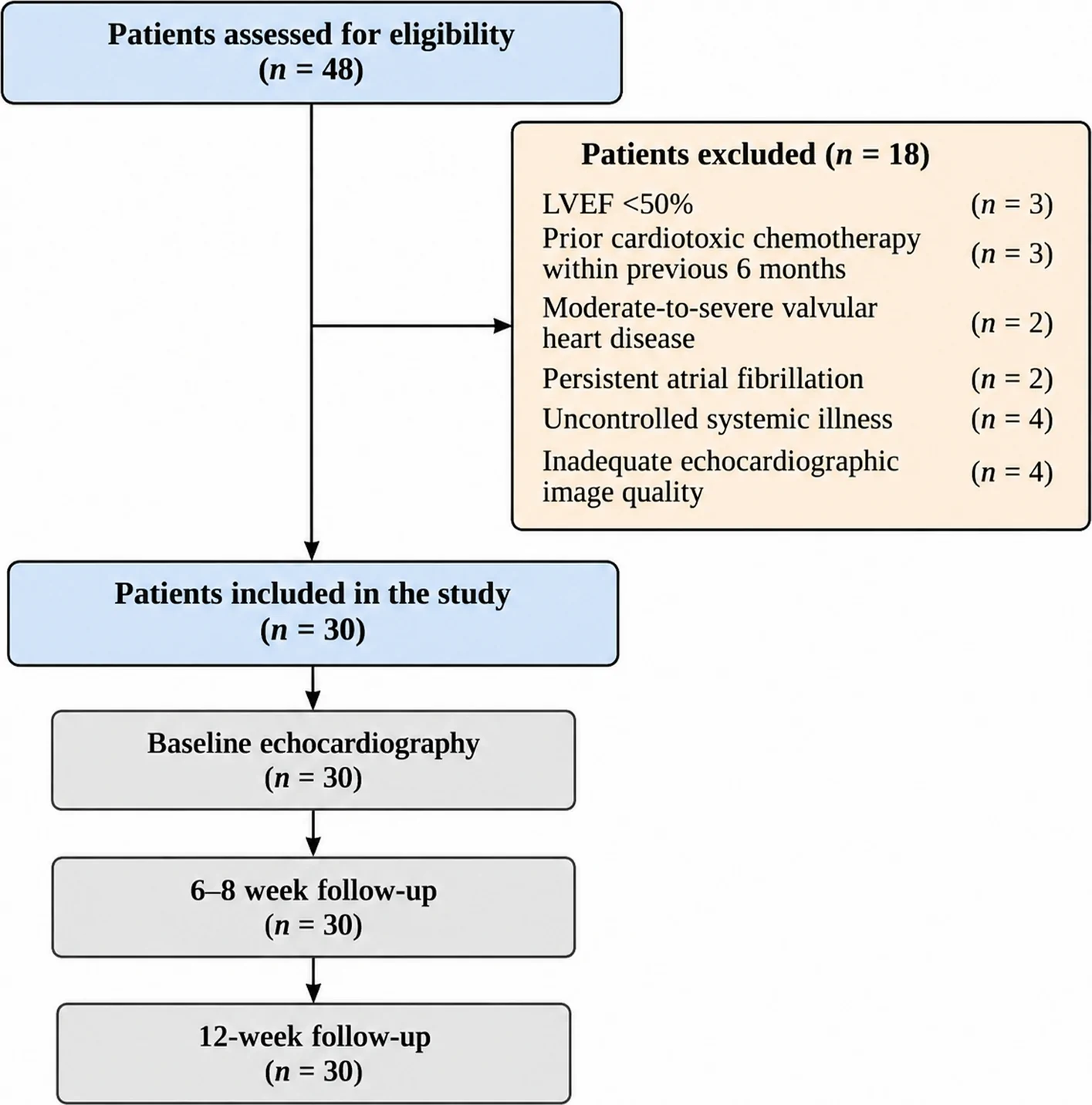
Flow diagram of patient selection and study follow-up

A total of 30 patients (mean age, 59.3 ± 11.2 years; 19 male patients, 63.3%) who initiated immune checkpoint inhibitor therapy were prospectively followed for 12 weeks. The patient selection process and study follow-up are summarized in Fig. 1.

The most common malignancies were non–small cell lung cancer (40.0%), melanoma (23.3%), and renal cell carcinoma (16.7%). Hypertension was present in 14 patients (46.7%), diabetes mellitus in 8 (26.7%), and prior coronary artery disease in 4 (13.3%). Baseline LVEF and GLS values were within normal limits in all participants (mean LVEF, 61.8% ± 4.5%; mean GLS, − 18.4% ± 2.3%), with no significant differences between patients who later developed cardiotoxicity and those who did not. Concomitant anticancer treatments, including chemotherapy, targeted therapy, and radiotherapy, were also recorded. No patient received anthracycline-based chemotherapy within the 6 months preceding ICI initiation. Baseline demographic, clinical and echocardiographic characteristics of the study population are presented in Table 1**.**

**Table 1:** Baseline demographic, clinical, and echocardiographic characteristics of the study population

Characteristic	Overall (*n* = 30)	Cardiotoxicity (*n* = 6)	No cardiotoxicity (*n* = 24)	*P*-value
Age (yr)	59.3 ± 11.2	61.5 ± 10.8	58.7 ± 11.4	0.52
Male sex	19 (63.3)	4 (66.7)	15 (62.5)	0.81
BMI (kg/m^2^)	27.6 ± 4.1	28.1 ± 3.9	27.5 ± 4.2	0.68
Hypertension	14 (46.7)	3 (50.0)	11 (45.8)	0.87
Diabetes mellitus	8 (26.7)	2 (33.3)	6 (25.0)	0.69
Hyperlipidemia	10 (33.3)	2 (33.3)	8 (33.3)	0.99
Current smoking	11 (36.7)	2 (33.3)	9 (37.5)	0.82
Coronary artery disease	4 (13.3)	1 (16.7)	3 (12.5)	0.74
Baseline heart rate (/min)	76 ± 11	79 ± 10	75 ± 11	0.39
Baseline SBP (mmHg)	128 ± 14	131 ± 15	127 ± 14	0.48
Baseline LVEF (%)	61.8 ± 4.5	61.2 ± 4.1	62.0 ± 4.6	0.63
Baseline GLS (%)	− 18.4 ± 2.3	− 18.2 ± 2.5	− 18.5 ± 2.2	0.78
High-sensitivity troponin (ng/L)	9.8 ± 4.2	10.9 ± 4.5	9.5 ± 4.1	0.44
NT-proBNP (pg/mL)	112 (65–198)	138 (92–244)	104 (60–176)	0.21
Non–small cell lung cancer	12 (40.0)	3 (50.0)	9 (37.5)	0.58
Melanoma	7 (23.3)	1 (16.7)	6 (25.0)	0.66
Renal cell carcinoma	5 (16.7)	1 (16.7)	4 (16.7)	0.99
Other malignancies	6 (20.0)	1 (16.7)	5 (20.8)	0.83
PD-1 inhibitor therapy	18 (60)	4 (66.7)	14 (58.3)	0.69
PD-L1 inhibitor therapy	8 (26.7)	1 (16.7)	7 (29.2)	0.52
CTLA-4–based combination	4 (13.3)	1 (16.7)	3 (12.5)	0.74

Values are presented as mean ± standard deviation, number (%), or median (interquartile range)

*BMI* body mass index, *CTLA-4* cytotoxic T-lymphocyte–associated antigen 4, *GLS* global longitudinal strain, *LVEF* left ventricular ejection fraction, *NT-proBNP* N-terminal pro–B-type natriuretic peptide, *PD-1* programmed cell death protein 1, *PD-L1* programmed death ligand 1, *SBP* systolic blood pressure

During the 12-week follow-up period, cardiotoxicity occurred in six patients (20.0%). Among these, three patients developed asymptomatic left ventricular systolic dysfunction meeting guideline-based cardiotoxicity criteria, two patients were clinically diagnosed with probable ICI-related myocarditis supported by biomarker elevation and imaging findings, and two patients experienced new-onset heart failure requiring temporary interruption of oncologic therapy. Two patients were classified as having probable ICI-associated myocarditis based on new cardiac troponin elevation accompanied by compatible clinical and imaging findings. Cardiac magnetic resonance imaging/endomyocardial biopsy findings were not performed in these patients. No cardiovascular deaths were observed.

Serial echocardiographic assessment demonstrated a more pronounced early deterioration in GLS among patients who developed cardiotoxicity. In this subgroup, GLS worsened from − 18.2% ± 2.5% at baseline to − 14.1% ± 2.8% at 6 to 8 weeks and further to − 13.2% ± 3.0% at 12 weeks. In contrast, LVEF remained relatively preserved at 6 to 8 weeks, decreasing from 61.2% ± 4.1% to 59.8% ± 4.7%, before declining to 55.5% ± 5.1% at 12 weeks. Among patients without cardiotoxicity, GLS changed from − 18.5% ± 2.2% at baseline to − 16.7% ± 2.2% at 6 to 8 weeks and − 15.6% ± 2.8% at 12 weeks, while LVEF changed from 62.0% ± 4.6% to 61.2% ± 4.5% and 59.5% ± 4.9%, respectively. These findings demonstrate a greater deterioration in GLS among patients who developed cardiotoxicity; however, because several cardiotoxicity events occurred before the scheduled 6- to 8-week assessment, the temporal relationship between GLS deterioration and cardiotoxicity cannot be interpreted as prospective in all patients. Serial echocardiographic changes, biomarker dynamics, and clinical outcomes during follow-up in patients receiving ICI therapy shown in Table 2.

**Table 2:** Serial echocardiographic changes, biomarker dynamics, and clinical outcomes during follow-up in patients receiving immune checkpoint inhibitor therapy (*n* = 30)

Parameter	Baseline	6–8 Weeks	12 Weeks	*P*-value
LVEF (%)				
Overall (*n* = 30)	61.8 ± 4.5	60.9 ± 4.6	58.7 ± 5.2	0.04
Cardiotoxicity (*n* = 6)	61.2 ± 4.1	59.8 ± 4.7	55.5 ± 5.1	0.03
No cardiotoxicity (*n* = 24)	62.0 ± 4.6	61.2 ± 4.5	59.5 ± 4.9	0.08
GLS (%)				
Overall (*n* = 30)	− 18.4 ± 2.3	− 16.2 ± 2.6	− 15.1 ± 3.0	< 0.001
Cardiotoxicity (*n* = 6)	− 18.2 ± 2.5	− 14.1 ± 2.8	− 13.2 ± 3.0	< 0.001
No cardiotoxicity (*n* = 24)	− 18.5 ± 2.2	− 16.7 ± 2.2	− 15.6 ± 2.8	0.01
Relative GLS reduction (%)	-	11.9 ± 6.8	17.8 ± 8.4	-
≥ 15% GLS reduction	0	7 (23.3)	7 (23.3)	-
Cardiotoxicity events	0	4 (13.3)	6 (20.0)	-
Symptomatic heart failure	0	1 (3.3)	2 (6.7)	-
Probable myocarditis	0	2 (6.7)	2 (6.7)	-
Ventricular arrhythmia	0	0	1 (3.3)	-
Troponin elevation	0	3 (10.0)	5 (16.7)	-
NT-proBNP increase > 30%	0	4 (13.3)	6 (20.0)	-

Values are presented as mean ± standard deviation or number (%). Cardiotoxicity was defined as an absolute ≥ 10% reduction in LVEF to < 50% and/or the occurrence of clinically significant cardiovascular events

*GLS* global longitudinal strain, *LVEF* left ventricular ejection fraction, *NT-proBNP* N-terminal pro–B-type natriuretic peptide

Biomarker measurements were available for most patients at each assessment. Troponin measurements were available in 28 patients (93.3%) at 6 to 8 weeks and 27 patients (90.0%) at 12 weeks; troponin elevation was observed in 3 (10.0%) and 5 patients (16.7%), respectively. NT-proBNP measurements were available in 27 patients (90.0%) at 6 to 8 weeks and 26 patients (86.7%) at 12 weeks, with a > 30% increase observed in 4 (13.3%) and 6 patients (20.0%), respectively. These biomarker abnormalities were observed more frequently during follow-up and accompanied the echocardiographic changes. However, because of the small number of cardiotoxicity events, a reliable subgroup comparison of troponin positivity between patients with and without cardiotoxicity was limited. Some patients experienced more than one cardiotoxicity manifestation during follow-up. The six patients meeting the composite cardiotoxicity endpoint were therefore classified according to their principal clinical presentation, while individual component events were recorded separately and were not considered mutually exclusive.

**Fig. 2:**
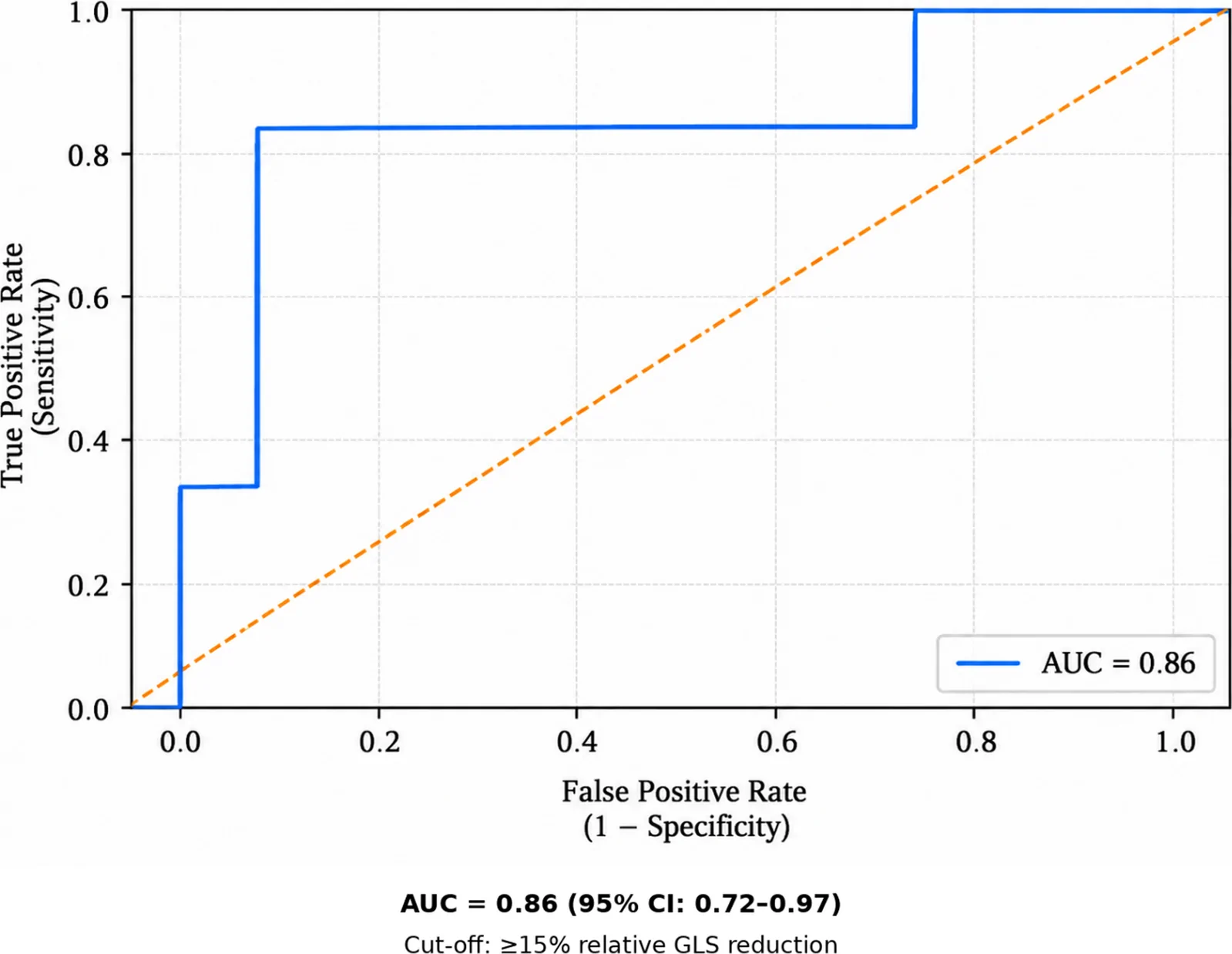
Receiver operating characteristic curve demonstrating the exploratory discriminatory ability of relative global longitudinal strain (GLS) reduction for cardiotoxicity in patients receiving immune checkpoint inhibitors. AUC, area under the curve; CI, confidence interval

A relative reduction in GLS of ≥ 15% from baseline was more frequent among patients with cardiotoxicity than among those without cardiotoxicity (83% vs. 9%, *P* < 0.001). In exploratory ROC analysis, the 6- to 8-week relative GLS reduction showed discriminatory ability for cardiotoxicity (AUC, 0.86; 95% CI, 0.72–0.97) (Fig. 2). The prespecified threshold of ≥ 15% GLS reduction yielded 83.3% sensitivity and 91.7% specificity. In exploratory logistic regression analysis, relative GLS reduction remained significantly associated with cardiotoxicity after adjustment for age, sex, hypertension, and cancer type (odds ratio, 7.4; *P* = 0.02). Given the small number of events and the temporal overlap between GLS assessment and cardiotoxicity in several patients, these analyses should be interpreted as exploratory associations rather than evidence of prospective prediction. Intraobserver and interobserver reproducibility of GLS measurements were excellent, with ICCs of 0.92 and 0.94 respectively.

## Discussion

In this prospective pilot study, we demonstrated that longitudinal deterioration in GLS occurs early after initiation of ICI therapy and precedes the development of conventional echocardiographic evidence of left ventricular systolic dysfunction. The principal finding of the present analysis is that a relative GLS reduction of ≥ 15% showed exploratory discriminatory ability for cardiotoxicity, whereas LVEF remained relatively preserved during the initial phase of follow-up. These results support the growing concept that myocardial deformation imaging provides superior sensitivity for detecting subclinical cardiac dysfunction in the emerging field of cardio-oncology, particularly in the context of immune-mediated myocardial injury.

ICI-associated cardiotoxicity represents a clinically significant yet incompletely characterized complication of modern oncologic therapy. Although myocarditis is considered the most severe manifestation, the clinical spectrum extends from asymptomatic myocardial inflammation and subtle contractile impairment to fulminant heart failure, malignant arrhythmias, and sudden cardiac death. Importantly, prior observational data suggest that ICI-related myocarditis may occur early after treatment initiation and is associated with higher mortality compared with other cancer therapy–related cardiac toxicities [4, 5, 11]. Therefore, identification of reliable, noninvasive markers capable of detecting myocardial involvement before irreversible injury develops is of substantial clinical relevance. Our findings align with this unmet need by demonstrating that GLS deterioration emerges within the first 6 to 8 weeks of therapy, a time window during which LVEF remains largely unchanged.

The limited sensitivity of LVEF for early myocardial injury is well established across multiple cardiotoxic cancer therapies, including anthracyclines and HER2-targeted agents. Because LVEF reflects global volumetric change rather than intrinsic myocardial fiber function, substantial myocardial damage may occur before a measurable decline becomes evident. In contrast, GLS directly quantifies longitudinal myocardial fiber shortening, which is particularly vulnerable to inflammatory and toxic injury. The present study extends this paradigm to ICI therapy, providing prospective evidence that strain-based imaging abnormalities precede overt systolic dysfunction in immune-mediated cardiotoxicity. This observation is mechanistically plausible, as immune activation, myocardial edema, and inflammatory infiltration are expected to impair myocardial deformation before chamber-level pump failure develops.

Another clinically meaningful observation in our cohort is the association between relative GLS reduction and cardiotoxicity. A ≥ 15% decline in GLS differentiated patients with and without cardiotoxicity and remained significantly associated with cardiotoxicity after adjustment for selected clinical variables. However, these findings should not be interpreted as evidence of prospective predictive value, because four of the six cardiotoxicity events occurred by the 6- to 8-week assessment and therefore the corresponding GLS measurements were obtained at or after the event. The observed discriminatory performance should consequently be regarded as exploratory and hypothesis-generating. In this pilot cohort, a ≥ 15% relative GLS reduction demonstrated good discriminatory performance for cardiotoxicity, although these findings should be interpreted cautiously given the small number of events. This threshold is consistent with prior strain-based definitions of subclinical cardiotoxicity used in chemotherapy populations, suggesting that deformation imaging may offer a unified framework for early cardiac surveillance across diverse oncologic therapies. From a practical standpoint, incorporation of GLS monitoring into routine echocardiographic follow-up of patients receiving ICIs could enable earlier cardiology referral, prompt biomarker testing, consideration of cardioprotective therapy, and individualized oncologic decision-making [6–8, 12].

Our findings should be interpreted in the context of previous studies evaluating myocardial strain during ICI therapy. Awadalla et al. demonstrated that GLS abnormalities may occur during ICI treatment and may be associated with subsequent cardiovascular events [13]. Similarly, Tamura et al. reported longitudinal changes in GLS in patients receiving ICIs [14]. More recent studies have also described deterioration in myocardial strain despite relatively preserved conventional measures of left ventricular systolic function. However, the literature is not entirely consistent, with some studies reporting no significant longitudinal change in left ventricular GLS. A recent systematic review of 12 studies including 554 ICI-treated patients similarly demonstrated overall deterioration in myocardial strain, while highlighting substantial heterogeneity between studies and the absence of significant GLS changes in some cohorts. Against this background, the contribution of the present study is not the demonstration that GLS can deteriorate during ICI therapy per se. Rather, our prospective protocol-driven design provides serial echocardiographic assessment at predefined early time points of 6 to 8 and 12 weeks after ICI initiation, allowing the temporal evolution of GLS to be examined alongside conventional echocardiographic parameters and cardiac biomarkers.

The absence of cardiovascular mortality in the present cohort should be interpreted cautiously given the modest sample size and short follow-up duration; however, the detection of cardiotoxicity in one-fifth of patients underscores the clinical relevance of systematic surveillance. Notably, most affected patients initially exhibited subclinical or mildly symptomatic disease, further emphasizing the importance of sensitive imaging tools. Early recognition may be particularly critical in ICI therapy because timely immunosuppression and temporary interruption of oncologic treatment have been associated with improved cardiac outcomes in myocarditis [15]. The absence of cardiovascular mortality in the present cohort should be interpreted cautiously given the modest sample size and short follow-up duration. The detection of cardiotoxicity in several patients highlights the potential relevance of systematic cardiovascular surveillance in patients receiving ICIs. However, whether serial GLS assessment can facilitate earlier therapeutic intervention, improve clinical decision-making, or reduce cardiovascular complications cannot be determined from the present study and requires evaluation in larger prospective studies.

Several limitations merit consideration. First, the study was conducted at a single center and included a relatively small, referral-based cohort of 30 patients. Therefore, selection and referral bias cannot be excluded, and the observed frequency of cardiotoxicity, including probable myocarditis, should not be interpreted as an estimate of its incidence in the broader ICI-treated population. The relatively high proportion of myocarditis cases should be considered hypothesis-generating and requires confirmation in larger multicenter cohorts. Second, the single-center design and relatively small sample size restrict generalizability and limit the power for multivariable modeling. Third, follow-up duration was confined to 12 weeks, precluding assessment of late cardiotoxicity or long-term cardiac recovery. Fourth, systematic cardiac biomarker and cardiac magnetic resonance imaging data were not available for all patients, which may have led to underrecognition of subclinical myocarditis. Biomarker measurements were not available at every scheduled assessment in all patients, limiting complete longitudinal comparisons of biomarker changes. Furthermore, given the heterogeneity of cancer types and concomitant oncologic treatments, a contribution of non-ICI–related cancer therapy to the observed cardiac abnormalities cannot be completely excluded, and causality between ICI exposure and cardiotoxicity should therefore be interpreted cautiously. The temporal relationship between GLS deterioration and cardiotoxicity warrants careful consideration. Because some cardiotoxicity events occurred before the scheduled 6–8-week echocardiographic assessment, GLS measurements obtained at or after the clinical diagnosis of cardiotoxicity cannot be interpreted as prospective predictors. Therefore, the observed relationship should be considered an early association unless the GLS measurement was confirmed to precede the cardiotoxicity event. Finally, although intraobserver reproducibility was excellent, inter-vendor variability of strain measurements remains a known methodological challenge in deformation imaging studies. Accordingly, the ROC and regression analyses should be considered exploratory assessments of association and discrimination rather than prospective prediction.

Despite these limitations, the prospective design, standardized imaging protocol, blinded strain analysis, and clinically adjudicated cardiotoxicity endpoints strengthen the validity of our findings. Importantly, this study adds to the limited prospective evidence regarding early cardiac surveillance in patients receiving ICIs and provides a basis for further prospective evaluation of serial GLS assessment in larger, multicenter cohorts.

## Conclusions

In this prospective pilot cohort of patients receiving ICI therapy, early changes in GLS were associated with cardiotoxicity despite relatively preserved LVEF. These findings are exploratory and hypothesis-generating and support further evaluation of serial GLS assessment in larger prospective studies. Whether GLS-guided surveillance can improve clinical management or cardiovascular outcomes remains to be determined.

## Data Availability

No datasets were generated or analysed during the current study.

## Abbreviations

AUC: Area under the curve
CI: Confidence intervals
CTLA-4: Cytotoxic T-lymphocyte–associated antigen 4
GLS: Global longitudinal strain
ICC: Intraclass correlation coefficient
ICI: Immune checkpoint inhibitor
LVEF: Left ventricular ejection fraction
NT-proBNP: N-terminal pro–B-type natriuretic peptide
PD-1: Programmed cell death protein 1
PD-L1: Programmed death ligand 1
ROC: Receiver operating characteristic
